# Accessible High-Throughput Virtual Screening Molecular Docking Software for Students and Educators

**DOI:** 10.1371/journal.pcbi.1002499

**Published:** 2012-05-31

**Authors:** Reed B. Jacob, Tim Andersen, Owen M. McDougal

**Affiliations:** Boise State University, Boise, Idaho, United States of America; Whitehead Institute, United States of America

## Abstract

We survey low cost high-throughput virtual screening (HTVS) computer programs for instructors who wish to demonstrate molecular docking in their courses. Since HTVS programs are a useful adjunct to the time consuming and expensive wet bench experiments necessary to discover new drug therapies, the topic of molecular docking is core to the instruction of biochemistry and molecular biology. The availability of HTVS programs coupled with decreasing costs and advances in computer hardware have made computational approaches to drug discovery possible at institutional and non-profit budgets. This paper focuses on HTVS programs with graphical user interfaces (GUIs) that use either DOCK or AutoDock for the prediction of DockoMatic, PyRx, DockingServer, and MOLA since their utility has been proven by the research community, they are free or affordable, and the programs operate on a range of computer platforms.

## Introduction

Advances over the past 20 years have made it feasible to use computationally intensive algorithms for high-throughput virtual screening (HTVS) and inverse virtual screening (IVS) of molecular interactions. HTVS involves docking many ligands against one or a few receptors, while IVS docks many receptors against one or a few ligands. A combination of pose identification and scoring algorithms constitute the foundation of docking engines, including DOCK [1] and AutoDock [2], [3]. Molecular docking results are evaluated by visual inspection of ligand pose or quantitatively using a scoring algorithm. Scoring algorithms may be incorporated into the docking engine, or accessed through third party software, such as Xscore and Medusa Score [4], [5]. Both Xscore and Medusa Score have been shown to have improved binding energy rankings over AutoDock when evaluated against a database of Protein Data Bank (pdb) benchmark standards. XScore is frequently cited as being used to re-rank AutoDock output and serves as the basis for AutoDock Vina [6]–[9].

DOCK and AutoDock were initially created during an era when computational resources for HTVS were prohibitively expensive and relatively primitive, but these programs have evolved over the years to be more user friendly, adaptable for HTVS, and useful as teaching and learning tools in a classroom setting. One noteworthy advance to AutoDock is a set of Python scripts and programs called MGLTools that facilitate and automate workflows required for the management of many simultaneous docking calculations. MGLTools contain a computer aided drug discovery (CADD) pipeline capable of accessing cloud resources for HTVS [10]. To enhance usability of DOCK and AutoDock, researchers have also developed graphical user interfaces (GUIs) that automate job management and submission for molecular docking calculation. The focus of this paper is HTVS GUI applications capable of processing large numbers of molecular interactions at an acceptable speed and cost, with reliable results, on a variety of computer platforms.

**Figure 1:**
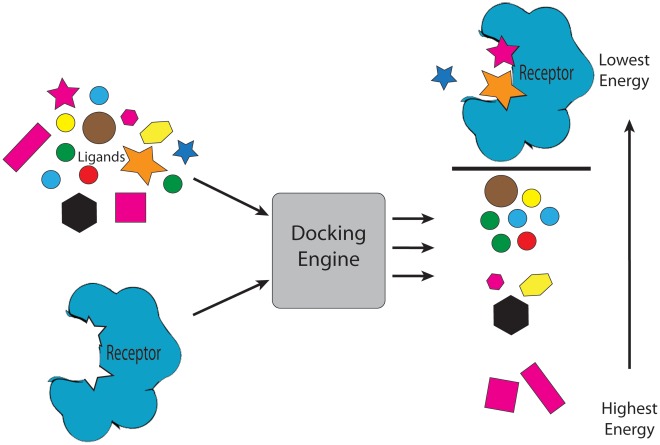
Depiction of high-throughput virtual screening: multiple ligands are docked to a receptor and ranked by energy estimate.

Docking engines calculate the free energy of binding (ΔG) between a ligand and a receptor, which is fundamental to the understanding of complex systems in biochemistry and molecular biology. The calculation of ΔG is based on estimates of the total energy of intermolecular forces of attraction including Van der Waals, hydrogen bonding, electrostatic, and hydrophobic. Ligands are ranked by the calculated ΔG value; lower ΔG values correspond to more favorable ligand binding, while higher ΔG values are less favorable. This gives teachers a rational and inexpensive tool for demonstrating to students how to assess and prioritize ligands for pursuit as drug targets (see Figure 1).

Molecular docking experiments involving either DOCK or AutoDock require an inordinate amount of time to set up, submit, compute, and analyze results. HTVS programs solve these problems through process automation. HTVS programs that use DOCK and AutoDock as their docking engines include DOVIS, VSDocker, WinDock, BDT, DockoMatic, PyRx, DockingServer, and MOLA. The HTVS programs we review are free or inexpensive, and can run on hardware ranging from a personal computer to a computing cluster. Cluster-based HTVS programs are DOcking-based VIrtual Screening (DOVIS) and Virtual Screening Docker (VSDocker), while WinDock and Blind Docking Tester (BDT) enable job queuing on only a single workstation. DockoMatic and Python Prescription (PyRx) can manage jobs independently of computer architecture, using a single workstation or cluster. DockingServer is a web-based application that runs independently of the user operating system, while MOLA can operate on networks consisting of heterogeneous computer architectures.

Educators can provide a visual context for the laboratory portion of their courses by selecting software programs described in this manuscript tailored to their computing capabilities. Open-access databases of receptor and ligand structures enable customized systems to be incorporated into the laboratory curriculum. Programs detailed in this manuscript were selected, in part, based on their use in solving research problems of instructional value and their relative ease of use in an educational environment.

## HTVS Programs Requiring Cluster Computing

### DOVIS and VSDocker

DOVIS and VSDocker are comprehensive HTVS programs that automate and enhance AutoDock. These programs can manage millions of docking experiments on large computing clusters, efficiently identifying and ordering the top scoring ligands [6]–[8]. DOVIS is Linux based, whereas VSDocker operates on Windows. Both programs rank and score results via user specified criteria. DOVIS contains a plug-in for third party scoring functions such as X-Score or Medusa Score [4], [5].

DOVIS has been used to screen hundreds of RNA aptamers for binding to gentamicin [11]. Aptamers are single-stranded RNA or DNA molecules, generally around 50 base pairs in length. Aptamers bind specific small ligands, such as amino-sugars, flavin, or peptides, and are significant as diagnostic molecules associated with gene regulation. DOVIS 2.0 is available for free download and it is an open-source program under the GNU General Public License [12].

VSDocker is designed to manage jobs using Windows XP or 2003 servers. VSDocker matches DOVIS in speed and performance, based on evaluation of molecular docking using ligands obtained from the ZINC database; run times were calculated to be 420 ligands/CPU/day [8], [13], [14]. VSDocker is free for non-commercial use but is not open source [8].

## HTVS Programs on Standalone Computer Systems

### WinDock

WinDock runs on a single Windows workstation. The docking engine for WinDock is DOCK. WinDock supports receptor homology model creation. Templates for receptors are identified via sequence alignment using ClustalX and T-coffee [15], [16]. WinDock then directs Modeller to construct a homology model [17]. WinDock includes a large 3D ligand library, or the user can access compounds of interest from their own ligand pdb database. Users can select force field, empirical, or knowledge-based ligand scoring algorithms to assess results [18]–[22].

WinDock has been used to study HIV-1 integrase enzyme binding to ligands isolated from three-Huang powder (THP), a Chinese medicinal formula [23]. Baicalein is one of approximately 16 components in THP and was shown to inhibit infectivity and replication of HIV by agonizing HIV-1 integrase. HIV-1 integrase consists of three domains, N-terminus, core, and C-terminus. WinDock identified the binding preference for baicalein to the middle of the ligand binding domain, the same site that was identified by co-crystallization with the inhibitor 5-CITEP [24]. A WinDock executable is available free of charge to students, academicians, and researchers by contacting the original author; the source code is not available [25].

### BDT

BDT is a Linux-based HTVS application that uses AutoDock to automate blind docking, inverse virtual screening, and ensemble docking studies [26]. BDT was used to study the binding of volatile anesthetic ligands, like halothane or sevoflurane, to amphiphilic pockets in volatile anesthetic binding proteins like serum albumin and apoferritin [27]. BDT was used to predict that Van der Waals forces were the predominant factor in the binding of volatile anesthetic ligands to compatible binding proteins. BDT is free for academic and non-commercial research purposes, though not open source [26], [27].

## HTVS Programs for Standalone or Cluster Computing

### DockoMatic

DockoMatic is a Linux-based HTVS program that uses a combination of front- and back-end processing tools for file preparation, result parsing, and data analysis [28]. DockoMatic can dock secondary ligands and may be used to perform inverse virtual screening [28], [29]. The DockoMatic GUI facilitates job creation, submission of jobs to AutoDock for docking, and result analysis for beginning and advanced users. The program can manage jobs on a single CPU or cluster, and generates ligand structure files by point mutation to an existing ligand pdb file or by entry of the single letter amino acid code for the peptide ligand sequence of interest.

DockoMatic has been used to study conotoxin binding to acetylcholine binding proteins (AchBPs) for drug design. AchBPs have similar homology to neuronal nicotinic acetylcholine receptors (nAchRs), which are pentameric ion channels responsible for the regulation of ions and small molecular neurotransmitters through biological membranes [30]. *Conus* snail venom peptides, specifically α-conotoxins (α-Ctxs), show targeted binding to both AchBPs and nAchRs. As a step to evaluate conotoxin binding nAchRs, a study was performed that looked at crystal structures of α-Ctxs bound to AchBPs. Conotoxin ligands that contained a public domain nuclear magnetic resonance (NMR) solution structure pdb file were analyzed in the bound state in the crystal structure, the peptide was removed from the ligand binding domain, and DockoMatic was used to redock the peptides. The peptides bound to AchBP included ImI[R11E], ImI[R7L], ImI[D5N], and PnIA[A10L:D14K]. The results demonstrated that DockoMatic may be used for computational prediction of peptide analog binding [28], [29]. DockoMatic is free, and open source, for academic and non-profit use and available at http://sourceforge.net/projects/dockomatic/.

### PyRx

PyRx runs on Windows, Mac OS X, or Unix/Linux computer clusters. PyRx can queue AutoDock jobs locally, or on a cloud using the Opal Web Services Toolkit [31], [32]. PyRx includes an embedded Python Molecular Viewer (ePMV) for visual analysis of results, as well as a built-in SQLite database for result storage [33].

PyRx has been used to study aromatase inhibitors (AIs). In post-menopausal women with breast cancer, increased levels of estrogen produced by the breast cancer cells increased cell production, creating a self-feedback loop [34], [35]. AIs have therapeutic value for patients that suffer breast cancer associated with excessive aromatase activity [34]. The AIs studied using PyRx had known crystal structures; PyRx output was compared to X-ray structures to validate computational binding prediction [34]. PyRx is free, open source, and distributed under the Simplified BSD license, and can be obtained from http://pyrx.sourceforge.net/downloads.

## Hardware-Independent HTVS Programs

### DockingServer

DockingServer is a comprehensive web service designed to make molecular docking accessible to all levels of users. DockingServer adds a MOZYME function, which uses atomic orbitals to calculate atomic charges, to its docking engine, AutoDock [36], [37]. The process for job submission is straightforward, and the output report gives the specific bond type interactions between each ranked result and the target receptor. A drawback is that the docking output structure files are large and DockingServer user storage space is limited. Thus, the number of parallel processes that can be run, prior to transferring or deleting files, is restricted.

DockingServer has been used to investigate human breast cancer resistance using a homology model of breast cancer resistant protein (BCRP) to characterize the potential interaction modes of the substrates mitoxantrone (MX), prazosin, Hoechst33342, and 7-Ethyl-10-hydroxycamptothecin (SN-38). Results indicated there is a central cavity in the middle of the lipid bilayer of BCRP capable of containing two substrates, instead of the previously hypothesized single substrate [38]. This study illustrates a possible mechanism for BCRP function that may lead to inhibitors for future drug development. The DockingServer web-based service is available for a modest annual subscription.

### MOLA

MOLA runs off a CD boot disk that preempts the local operating system with its own operating system [39]. MOLA is capable of configuring a temporary computer cluster from heterogeneous, networked standalone computers, regardless of operating platform. This program is intended for research labs without access to a dedicated computer cluster. MOLA includes AutoDock Tools (ADT), which is a program included within MGLTools, for grid parameter file (gpf) creation and ligand/receptor preparation. ADT also generates an analysis spreadsheet ranked by the lowest binding energy and distance to the active site [10]. MOLA does require some familiarity with ADT and preparation of receptor files for AutoDock submission.

MOLA was used to investigate ligand binding to retinol binding protein, HIV-1 protease, and trypsin-benzamide, each with a ligand library search of over 500 ligands and decoys, recreating the approximate potential bell curve of these ligand sets to each receptor. MOLA is a free download as an image file for direct burning to disk [39]. The source code is not available.

## Discussion

The role of computational molecular docking in the educational and research community is evolving at a rapid rate. Access to this field by an ever increasing number of students, teachers, and scientists has been facilitated by software programs similar to those described here. Each program we describe has been used to address real world research problems that educators may find instructive for students. Table 1 summarizes the features of each HTVS program reviewed. Instructors should select a program to use in their courses dependent upon their curriculum, computer hardware access, financial resources, and desired instructional objectives.

**Table 1:** An overview of features for HTVS programs with GUIs.

	WinDock	BDT	Dovis	VS Docker	DockoMatic	DockingServer	PyRx	MOLA
Platform	Windows	Linux	Linux	Windows	Linux	Web	Linux, Unix, Windows, Mac OS X	All
Release Date	2007	2006	2008	2010	2010	2009	2009	2010
Docking Engine	DOCK	AutoDock	AutoDock	AutoDock	AutoDock	AutoDock	AutoDock	AutoDock
Reference	[25]	[26]	[6], [7]	[8]	[28], [29]	[36], [37]	[31]	[39]
Homology Modeling	√							
Ligand Library	√	√	√	√	√	√	√	√
Ligand Creation					√	√		
Open Source			√	√	√		√	
Cluster/Cloud			√	√	√		√	√
Installer^a^	√			√		N/A	√	√
Local Resource Demand^b^	S	S	M	M	E	S	E	E
Documentation^c^	1	1	2	2	1	5	3	2
Ease of Use^d^	1	1	3	3	2	1	3	4

Programs appear across the top and features down the left side of the table.

a If an N/A appears that program needs no installer; it is a web interface.

b S, minimum program requirement is a single computer workstation; M, multiple computers in a cluster are required; and E, single or multi-processor enabled.

c Rated on a 1–5 scale with 1 being basic installation instructions to 5 being in depth tutorials and worked examples for applications.

d Rated on a 1–5 operator scale with 1 being a user with basic computer skills to 5 being an experienced programmer.

The HTVS programs described in this manuscript were developed with the common goal of enhancing the ability to perform molecular docking studies using one of two well-established docking engines, DOCK or AutoDock. The optimal program for use to explain biological principles to students is dependent on the specific goals of the instructor. For a class in a department with limited computer availability interested in occasional docking investigations, we suggest WinDock or PyRx, as both programs are available for a Windows operating system. For more in-depth docking studies with Linux operating system availability, BDT, PyRx, and DockoMatic may be preferable. If a Linux cluster is available, then DockoMatic, DOVIS, or PyRx are recommended, or VSDocker for a Windows cluster. If an instructor has access to multiple networked computers, without a cluster, MOLA is ideal for HTVS. For instructors with limited computer resources, DockingServer is an external web service for a reasonable subscription. Of these programs, DOVIS, VSDocker, and BDT provide rank ordered lists of results, with limited capacity for the user to visualize the docked molecules without accessing another software program like PyMol. For result visualization, DockoMatic and MOLA provide a link directly to PyMol and ADT, respectively [40], [41]. WinDock, PyRx, and DockingServer contain fully integrated visualization capabilities for all steps in the process of docking to result analysis.

In addition to computational requirements, each HTVS program has unique features to assist in docking studies and data analysis. BDT is optimal if the instructor presents students with a project to study a specific receptor that does not have a known binding pocket. If the instructor requires construction of homology models, WinDock contains a Modeller interface. If the primary instructional goal is limited to screening ligands, then DOVIS or VSDocker work well. To study point mutations of small cyclic peptides like conotoxins or other peptide ligands, then DockoMatic with automated peptide analog structure creation is a recommended option. PyRx is useful for ligand comparison studies because it offers well-integrated storage and visualization of HTVS results that facilitate binding analysis. For those new to the field of computational chemistry, DockingServer is a comprehensive, user friendly, and supported program.

## References

[1] Kuntz ID, Blaney JM, Oatley SJ, Langridge R, Ferrin TE (1982). A geometric approach to macromolecule-ligand interactions.. J Mol Biol.

[2] Morris G, Goodsell D, Halliday R, Huey R, Hart W (1998). Automated docking using a Lamarckian genetic algorithm and an empirical free energy function.. J Comput Chem.

[3] Goodsell DS, Olson AJ (1990). Automated docking of substrates to proteins by simulated annealing.. Proteins.

[4] Wang R, Lai L, Wang S (2002). Further development and validation of empirical scoring functions for structure-based binding affinity prediction.. J Comput-Aided Mol Des.

[5] Yin S, Biedermannova L, Vondrasek J, Dokholyan NV (2008). MedusaScore: an accurate force field-based scoring function for virtual drug screening.. J Chem Inf Model.

[6] Jiang X, Kumar K, Hu X, Wallqvist A, Reifman J (2008). DOVIS 2.0: an efficient and easy to use parallel virtual screening tool based on AutoDock 4.0.. Chem Cent J.

[7] Zhang S, Kumar K, Jiang X, Wallqvist A, Reifman J (2008). DOVIS: an implementation for high-throughput virtual screening using AutoDock.. BMC Bioinformatics.

[8] Prakhov ND, Chernorudskiy AL, Gainullin MR (2010). VSDocker: a tool for parallel high-throughput virtual screening using AutoDock on Windows-based computer clusters.. Bioinformatics.

[9] Trott O, Olson AJ (2010). Software news and update AutoDock Vina: improving the speed and accuracy of docking with a new scoring function, efficient optimization, and multithreading.. J Comput Chem.

[10] Sanner MF (1999). Python: a programming language for software integration and development.. J Mol Graph Model.

[11] Chushak Y, Stone MO (2009). In silico selection of RNA aptamers.. Nucleic Acids Res.

[12] Jiang X, Kumar K, Hu X, Wallqvist A, Reifman J (2008). DOVIS, version 2.0.. http://www.bioanalysis.org/downloads/DOVIS-2.0.1-installer.tar.gz.

[13] Irwin JJ, Shoichet BK (2004). ZINC – A free database of commercially available compounds for virtual screening.. J Chem Inf Model.

[14] Collignon B, Schulz R, Smith JC, Baudry J (2011). Task-parallel message passing interface implementation of Autodock4 for docking of very large databases of compounds using high-performance super-computers.. J Comp Chem.

[15] Thompson JD, Higgins DG, Gibson TJ (1994). CLUSTAL W: improving the sensitivity of progressive multiple sequence alignment through sequence weighting, position-specific gap penalties and weight matrix choice.. Nucleic Acids Res.

[16] O'Sullivan O, Suhre K, Abergel C, Higgins DG, Notredame C (2004). 3DCoffee: combining protein sequences and structures within multiple sequence alignments.. J Mol Biol.

[17] Sali A, Blundell T (1993). Comparative protein modelling by satisfaction of spatial restraints.. J Mol Biol.

[18] Muegge I, Martin YC (1999). ARTICLES - a general and fast scoring function for protein – ligand interactions: a simplified potential approach.. J Med Chem.

[19] Wang R, Liu L, Lai L, Tang Y (1998). SCORE: a new empirical method for estimating the binding affinity of a protein-ligand complex.. J Mol Model.

[20] Eldridge MD, Murray CW, Auton TR, Paolini GV, Mee RP (1997). Empirical scoring functions: I. The development of a fast empirical scoring function to estimate the binding affinity of ligands in receptor complexes.. J Comput-Aided Mol Des.

[21] Böhm HJ (1994). The development of a simple empirical scoring function to estimate the binding constant for a protein-ligand complex of known three-dimensional structure.. J Comput-Aided Mol Des.

[22] Zhang C, Liu S, Zhu Q, Zhou Y (2005). A knowledge-based energy function for protein–ligand, protein–protein, and protein–DNA complexes.. J Med Chem.

[23] Hu JZ, Bai L, Chen DG, Xu QT, Southerland WM (2010). Computational investigation of the anti-HIV activity of Chinese medicinal formula three-Huang powder.. Interdiscip Sci.

[24] Goldgur Y, Craigie R, Cohen GH, Fujiwara T, Yoshinaga T (1999). Structure of the HIV-1 integrase catalytic domain complexed with an inhibitor: a platform for antiviral drug design.. Proc Natl Acad Sci U S A.

[25] Hu Z, Southerland W (2007). WinDock: structure-based drug discovery on Windows-based PCs.. J Comput Chem.

[26] Vaqué M, Arola A, Aliagas C, Pujadas G (2006). BDT: an easy-to-use front-end application for automation of massive docking tasks and complex docking strategies with AutoDock.. Bioinformatics.

[27] Streiff JH, Jones KA (2008). Volatile anesthetic binding to proteins is influenced by solvent and aliphatic residues.. J Chem Inf Model.

[28] Bullock CW, Jacob RB, McDougal OM, Hampikian G, Andersen T (2010). Dockomatic - automated ligand creation and docking.. BMC Research Notes.

[29] Jacob RB, Bullock CW, Andersen T, McDougal OM (2011). DockoMatic: Automated peptide analog creation for high throughput virtual screening.. J Comp Chem.

[30] Albuquerque EX, Alkondon M, Pereira EFR, Castro NG, Schrattenholz A (1997). Properties of neuronal nicotinic acetylcholine receptors: Pharmacological characterization and modulation of synaptic function.. J Pharmacol Exp Ther.

[31] Wolf LK (2009). digital briefs: New software and websites for the chemical enterprise.. C&EN.

[32] Ren J, Williams N, Clementi L, Krishnan S, Li WW (2010). Opal web services for biomedical applications.. Nucleic Acids Res.

[33] Johnson Graham T, Autin L, Goodsell David S, Sanner Michel F, Olson AJ (2011). ePMV embeds molecular modeling into professional animation software environments.. Structure (London, England : 1993).

[34] Suvannang N, Nantasenamat C, Isarankura-Na-Ayudhya C, Prachayasittikul V (2011). Molecular docking of aromatase inhibitors.. Molecules.

[35] Fontham ETH, Thun MJ, Ward E, Balch AJ, Delancey JOL, Samet JM (2009). American Cancer Society perspectives on environmental factors and cancer.. CA Cancer J Clin.

[36] Virtua Drug Ltd (2009). DockingServer.. http://www.dockingserver.com.

[37] Bikadi Z, Hazai E (2009). Application of the PM6 semi-empirical method to modeling proteins enhances docking accuracy of AutoDock.. J Cheminf.

[38] Cai X, Bikadi Z, Ni Z, Lee E-W, Wang H (2010). Role of basic residues within or near the predicted transmembrane helix 2 of the human breast cancer resistance protein in drug transport.. J Pharmacol Exp Ther.

[39] Abreu R, Froufe H, Queiroz M, Ferreira I (2010). MOLA: a bootable, self-configuring system for virtual screening using AutoDock4/Vina on computer clusters.. J Cheminf.

[40] Shrodinger LLC (2009). The PyMOL molecular graphics system. Version 1.3 ed.. http://www.pymol.org.

[41] Li C, Xu L, Wolan D, Wilson L, Olson A (2004). AutoDock Tools: virtual screening of human 5-aminoimidazole-4-carboxamide ribonucleotide transformylase against the NCI diversity set by use of AutoDock to identify novel nonfolate inhibitors.. J Med Chem.

